# Tetraspanin 8-Rictor-Integrin α3 Complex Is Required for Glioma Cell Migration

**DOI:** 10.3390/ijms16035363

**Published:** 2015-03-09

**Authors:** Si-Jian Pan, Shi-Kun Zhan, Yi-Xin Pan, Wei Liu, Liu-Guan Bian, Bomin Sun, Qing-Fang Sun

**Affiliations:** 1 Department of Neurosurgery, Rui Jin Hospital, School of Medicine, Shanghai Jiao Tong University, 197 Shanghai Ruijin Second Road, Shanghai 200025, China; E-Mails: pansijian163@163.com (S.-J.P.); blg11118@rjh.com.cn (L.-G.B.); 2 Department of Stereotactic and Functional Neurosurgery, Rui Jin Hospital, School of Medicine, Shanghai Jiao Tong University, 197 Shanghai Ruijin Second Road, Shanghai 200025, China; E-Mails: shikun_zhan@163.com (S.-K.Z.); yixinpan@163.com (Y.-X.P.); doctorliuwei@163.com (W.L.); bomin_sun@163.com (B.S.)

**Keywords:** malignant glioma, tetraspanin 8, integrin α3, rictor, mTORC2, AKT and cell migration

## Abstract

The malignant glioma remains one of the most aggressive human malignancies with extremely poor prognosis. Glioma cell invasion and migration are the main causes of death. In the current study, we studied the expression and the potential functions of tetraspanin 8 (Tspan8) in malignant gliomas. We found that Tspan8 expression level is high in both malignant glioma tissues and in several human glioma cell lines, where it formed a complex integrin α3 and rictor, the latter is a key component of mammalian target of rapamycin (mTOR) complex 2 (mTORC2). Disruption of this complex, through siRNA-mediated knockdown of anyone of these three proteins, inhibited U251MG glioma cell migration *in vitro*. We further showed that Tspan8-rictor association appeared required for mTORC2 activation. Knockdown of Tspan8 by the targeted siRNAs prevented mTOR-rictor (mTORC2) assembly as well as phosphorylation of AKT (Ser-473) and protein kinase C α (PKCα) in U251MG cells. Together, these results demonstrate that over-expressed Tspan8 in malignant glioma forms a complex with rictor and integrin α3 to mediate mTORC2 activation and glioma cell migration. Therefore, targeting Tspan8-rictor-integrin α3 complex may provide a potential therapeutic intervention for malignant glioma.

## 1. Introduction

The malignant glioma remains one of the most aggressive human malignancies with extremely poor prognosis, and its overall survival is quite low (6–12 months) [1]. The poor prognosis is due to many reasons, including late diagnosis, absence of specific diagnostic markers, resistance of traditional therapy (*i.e.*, radiation and chemotherapy), and most importantly, the high invasion and migration potential of the tumor cells [2,3]. Clinically, temozolomide (TMZ) is the only approved chemo-drug for malignant glioma, which only showed limit pro-surviving value [2]. Several TMZ-based combine regimens are being tested in clinical trials with only limited values [2]. Tumor cell infiltration and local invasion remain the overwhelming causes of death for malignant glioma cancer patients. To establish effective therapeutic methods, new targets implied in these processes have to be identified [4].

Tetraspanins, a family of four transmembrane proteins, were shown to physically associate with a large variety of transmembrane and/or cytosolic proteins, and regulate many important cellular functions, such as cell migration, adhesion, invasion and proliferation [5,6,7]. Tetraspanins regulate, stabilize or inhibit activities of associated molecule [6]. For example, tetraspanins, in complex with various integrins, are important for integrin compartmentalization, internalization, recycling and signaling, as well as cell spreading, migration and local invasion [8,9,10].

Recent studies have shed lights on the functions of tetraspanins in cancer progression [11,12,13]. Several tetraspanins are over-expressed in many cancers, and are often associated with poor prognosis [11,12]. Tetraspanin 8 (Tspan8), a tetraspanin that is uniquely expressed only in a small number of normal tissues, is also found over-expressed in several cancers including colon, liver, prostate, ovarian and cervical cancers [14]. High Tspan8 expression in colorectal cancer often indicate poor prognosis [15]. Further, a mouse monoclonal antibody against Tspan8 could suppress colorectal cancer HT-29 xenograft *in vivo* growth in nude mice [14]. Over-expression of this molecule was also seen in esophageal carcinoma [16], hepatocellular carcinoma [17] and melanoma [18], reregulating cancer cell progression.

Rictor, a novel binding partner of mammalian target of rapamycin (mTOR), is an indispensable component of mTOR complex 2 (mTORC2) [19,20]. Rictor-mTOR complex modulates the actin cytoskeleton through phosphorylation of protein kinase C α (PKCα) and AKT (Ser-473) [19,20]. Rictor is important for cell migration [19,20]. In gliomas, overexpression of rictor causes mTORC2 overactivation to increase cell motility [21]. The underlying mechanism of rictor-mTORC2 activation in gliomas has not been fully understood.

In the current study, we found that Tspan8 is over-expressed in human malignant glioma tissues, as well as in several human glioma cell lines. Over-expressed Tspan8 forms a complex with rictor and integrin α3, which is required for mTORC2 activation and glioma cell migration.

## 2. Results

### 2.1. Over-Expression of Tspan8 in Human Malignant Glioma Tissues and Cell Lines

**Figure 1:**
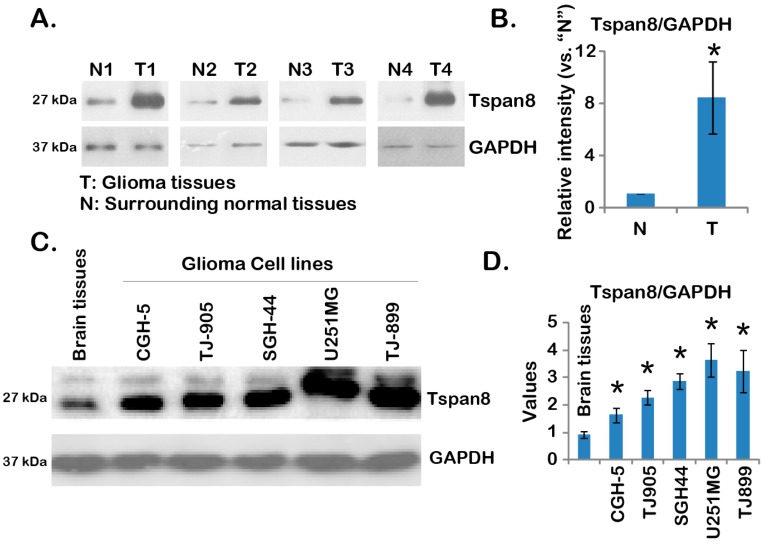
Over-expression of tetraspanin 8 (Tspan8) in human malignant glioma tissues and cell lines. Expression of Tspan8 and glyceraldehyde-3-phosphate dehydrogenase (GAPDH) (the loading control) in four different human malignant glioma tissues (“T”) or corresponding surrounded normal brain tissues (“N”) was tested by Western blots (**A**); relative Tspan8 expression (*vs.* GAPDH) was quantified (**B**); Expression of Tspan8 and GAPDH in normal brain tissues (from stroke patient) or in different human glioma cell lines (CGH-5, TJ-899, SGH-44, TJ-905 and U251MG) was shown (**C**); relative Tspan8 expression (*vs.* GAPDH) was quantified (*n* = 3, **D**). Data were presented as mean ± SD (**B**). * *p* < 0.05 *vs.* surrounded normal brain tissues (“N”) (**B**). Experiments in this and all following figures were repeated three times, with similar results obtained.

This study is set to test the expression and potential functions of tetraspanin 8 (Tspan8) in malignant gliomas. Through Western blot, we examined the expression of Tspan8 in human malignant gliomas. As demonstrated, the expression level of this molecule in malignant gliomas (“T”) is significantly higher compared to that in normal surrounding brain tissues (“N”) (Figure 1A). It is about 6–7 times more Tspan8 expression in malignant gliomas than that in normal tissues (Figure 1B). Note that all the tested malignant gliomas were grade 3–4. Tspan8 was also over-expressed in multiple human glioma cell lines, as compared to normal brain tissues (Figure 1C,D). Among all the cell lines tested, U251MG appeared to have most Tspan8 (Figure 1C,D). Together, these results demonstrated over-expression of Tspan8 in human malignant glioma tissues and cell lines.

### 2.2. Tspan8 Associates with Rictor and Integrin α3 in both Human Glioma Tissues and Cell Lines

**Figure 2:**
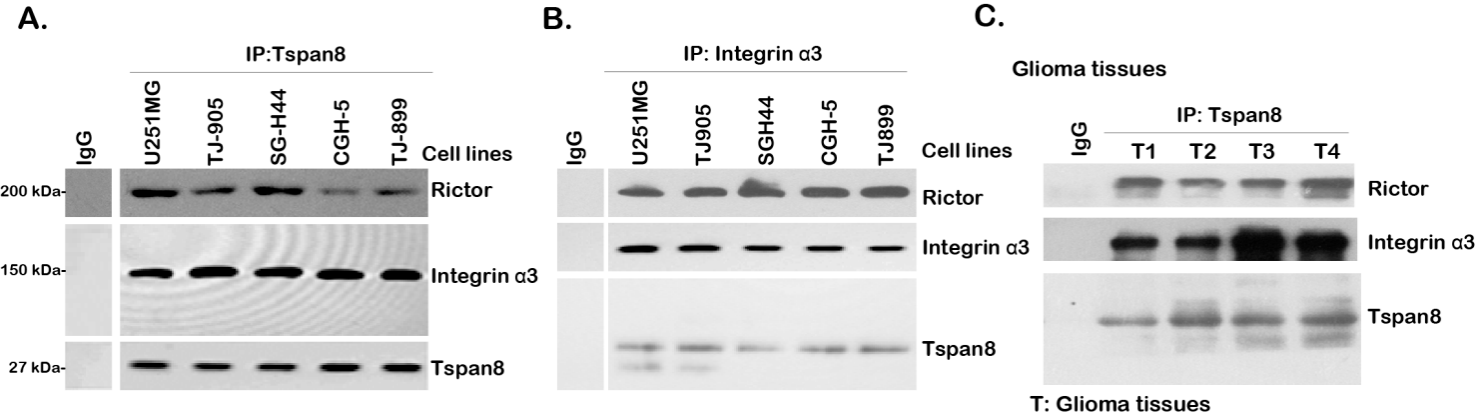
Tspan8 associates with rictor and integrin α3 in both human glioma tissues and glioma cell lines. The association between Tspan8, integrin α3 and rictor in multiple human glioma cell lines (CGH-5, TJ-899, SGH-44, TJ-905 and U251MG) (**A**,**B**) or in four different human glioma tissue lysates (**C**) was tested by Co-Immunoprecipitation (Co-IP) as described.

Tetraspanins could form complexes with various integrins and many other transmembrane and/or cytosolic proteins to regulate cell migration and several other important cellular functions [5,6,7]. Here, using the Co-IP assay, we found that Tspan8 formed a complex with integrin α3 in the tested human glioma cell lines (CGH-5, TJ-899, SGH-44, TJ-905 and U251MG) (Figure 2A,B). Significantly, rictor, an irreplaceable component of mTOR complex 2 (mTORC2) [22,23], was also in the complex of Tspan8-integrin α3, indicating a possible role of Tspan8 in mTORC2 activation. Further, as shown in Figure 2C, Tspan8-integrin α3-rictor complex was also seen in human glioma tissues (Figure 1). Together, these results show that over-expressed Tspan8 forms a complex with integrin α3 and rictor in human glioma tissues and cell lines.

### 2.3. SiRNA-Mediated Knockdown of Tspan8 or Integrin α3 Inhibits U251MG Cell Migration

**Figure 3:**
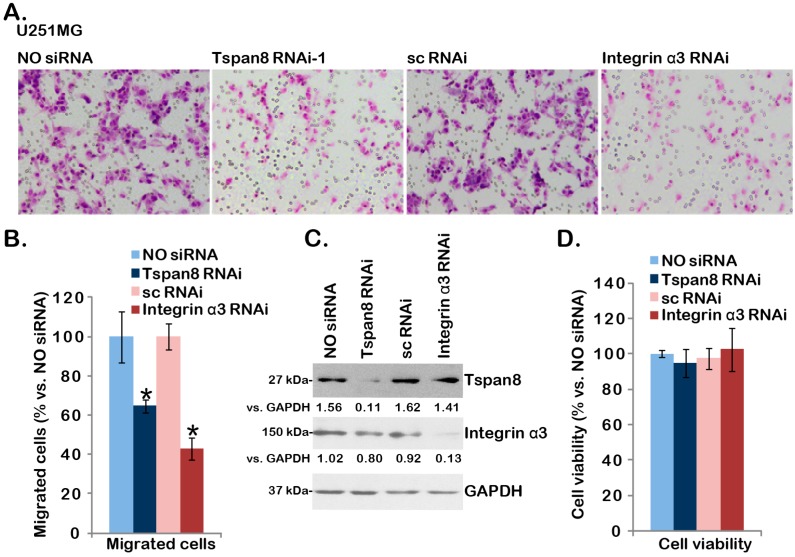
SiRNA-mediated knockdown of Tspan8 or integrin α3 inhibits U251MG cell migration. *In vitro* migration of U251MG cells transfected with or without indicated siRNA (100 nM, 48 h) was tested by “transwell” assay described (**A**, 18 h); the number of migrated cells was counted and normalized to the control U251MG cells (“NO siRNA”) (**B**); Expression of Tspan8, integrin α3 and GAPDH in above U251MG cells was shown in (**C**); Above U251MG cells were also subjected to MTT cell viability assay, and OD values were normalized to the control U251MG cells (**D**, 18 h). Magnification: 1:100 (**A**). Data were presented as mean ± SD. * *p* < 0.05 *vs.* control U251MG cells.

Next, we tested the potential function of this complex in U251MG cells. The “transwell” migration assay results in Figure 3A demonstrated that siRNA-mediated knockdown of Tspan8 or integrin α3 dramatically inhibited U251 cell in vitro migration, while scramble control siRNA (“sc RNAi”) had no such effect. The number of migrated U251MG cells with Tspan8 or integrin α3 RNAi was significantly lower than that of control U251MG cells (“NO siRNA”) or U251MG cells transfected with sc RNAi (Figure 3B). Western blot results in Figure 3C displayed RNAi efficiency, and over 80% of targeted protein was downregulated by corresponding siRNA (see Figure 3C, quantification). Results in Figure 3D showed that the viability of U251MG cells was not affected by Tspan8 or integrin α3 siRNA, indicating that cell migration inhibition by knocking-down of Tspan8 or integrin α3 was unlikely due to differences in cell proliferation or cell survival.

### 2.4. Tspan8-Rictor Association Is Required for mTORC2 Activation, AKT Ser-473 Phosphorylation and U251MG Cell Migration

**Figure 4:**
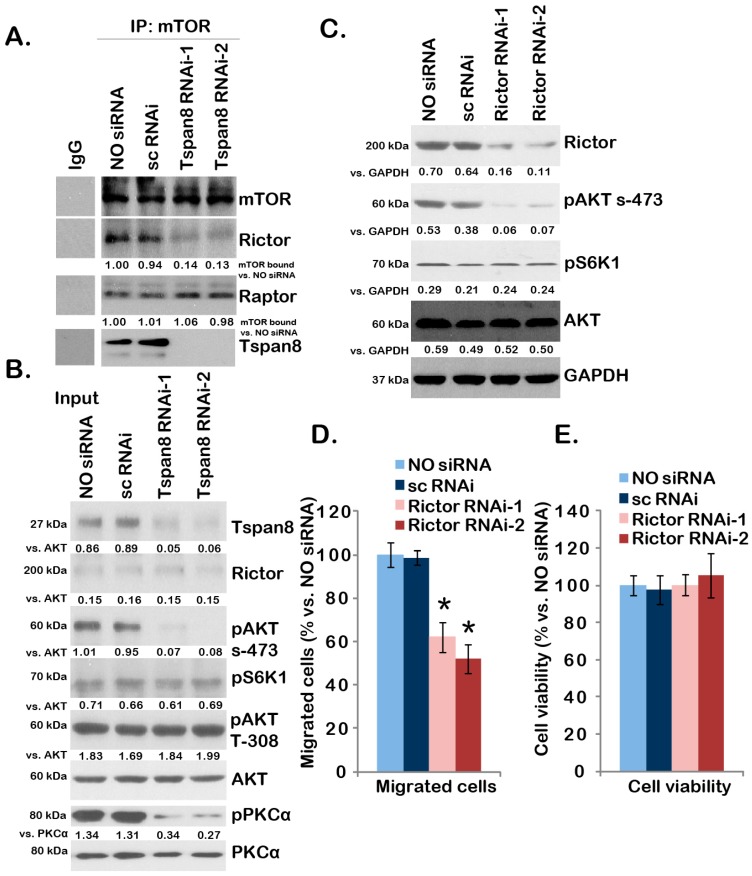
Tspan8-rictor association is required for mTORC2 activation, AKT Ser-473 phosphorylation and U251MG cell migration. The mTOR-rictor-Raptor-Tspan8 association in control U251MG cells (NO siRNA) or U251MG cells with indicated siRNA (sc-RNAi, Tspan8-siRNA-1 or Tspan8-siRNA-2, 100 nM, 48 h) was tested by Co-IP (**A**); Expression of listed proteins was tested by Western blots as “Input” (**B**); U251MG cells transfected with or without indicated siRNA (sc-RNAi, rictor siRNA-1 or rictor siRNA-2) were subjected to “Transwell” assay (**D**, 18 h); and MTT cell viability assay (**E**, 18 h); expression of listed protein in above cells was also shown (**C**). Data were presented as mean ± SD. * *p* < 0.05 *vs.* control U251MG cells.

Above results showed that rictor, the indispensable component of mTORC2 [19], was in the complex with Tspan8, we thus tested the role of Tspan8 in mTORC2 activation in U251MG cells. The Co-IP assay results in Figure 4A showed that knockdown of Tspan8 by siRNA disrupted the mTOR-rictor association (mTORC2 assembly), but not the mTOR-Raptor mTORC1 assembly [23]. Phosphorylation of PKCα (Ser-657) and AKT Ser-473, the indicators of mTORC2 activation [20], was also reduced in Tspan8 silenced U251MG cells (Figure 4B); While S6K1 phosphorylation, an indicator of mTORC1 activation, as well as AKT Thr-308 phosphorylation were not affected by Tspan8 siRNAs in U251MG cells (Figure 4B). Note that two non-overlapping Tspan8 siRNAs were applied, each one of them showed similar results (Figure 4A,B). As expected, rictor knockdown by targeted siRNAs also prevented AKT Ser-473 phosphorylation, but not S6K1 phosphorylation in U251MG cells (Figure 4C). Phosphorylation of PKCα was also inhibited by rictor siRNA (Data not shown). Significantly, U251 cell *in vitro* migration, tested by transwell assay, was suppressed by two non-overlapping rictor siRNAs (Figure 4D). Again, the cell viability (18 h period) was not affected by rictor siRNA knockdown (Figure 4E), indicating that the reduced migration seen in rictor-silenced cells was not likely due to cell proliferation variations. Together, these results indicate that Tspan8-rictor complex might be important for mTORC2 assembly and activation as well as glioma cell migration.

## 3. Discussion

Although the role of Tspan8 or other tetraspanins in cell migration has been proposed [13,18], the underlying mechanisms of tetraspanins promoting cell migration have not been fully understood [24,25,26,27]. Tspan8 was shown to promote cancer cell motility through interaction with several integrins, including α6β4, α3β1 and α6β1 integrins [24,25,26,27]. Here we showed that Tspan8 was over-expressed in many human glioma tissues and cell lines. One important finding of this study is that Tspan8 was in the complex with α3 integrin in human malignant glioma tissues or cells. The fact that siRNA knockdown of Tspan8 or α3 integrin inhibited U251MG cell *in vitro* migration suggesting that Tspan8 might promote invasive behaviors of glioma cells through complex with α3 integrin. As a matter of fact, α3 integrin was known to play vital roles in migration and invasion of malignant glioma cells [28,29].

There are at least two mTOR complexes that have been indentified thus far: namely mTORC1 and mTORC2 [30,31]. Both complexes were shown to play significant roles in glioma initiation and progression [30,31]. Traditional mTORC1 is composed of mTOR, Raptor, mLST8, as well as PRAS40 and DEPTOR [22]. Activated AKT phosphorylates and inhibits TSC2, hampering TSC2 from inhibiting mTORC1, leading to mTORC1 activation and 4E-BP-1/S6K phosphorylation [22]. MTORC1 regulate many cellular functions that are required for cancer progression, such as protein translation, energy metabolism and cell growth [22,23]. On the other hand, the recently discovered mTORC2 contains mTOR, rictor, Sin1 and possible several others. MTORC2 is the upstream kinase of AKT Ser-473 and several other AGC kinases [22,23]. In this study, we found that Tspan8 might also be involved in mTORC2 activation in glioma cells.

The role of mTORC2 in cell invasion or migration has been extensively studied [21]. MTORC2 is known to phosphorylate AGC protein kinases (*i.e.*, protein kinase C (PKC)) to regulate cell migration [32]. Kim *et al.* [33] showed that rictor silencing inhibited ovarian cancer cell migration caused by a constitutively-active AKT. In the current study, we found that rictor knockdown significantly suppressed U251MG cell *in vitro* migration, further supporting a key role of mTORC2 in glioma cell migration.

The upstream signaling of mTORC2 activation has not been fully understood [34]. Here, we showed that Tspan8 might serve as the potential upstream signaling for mTORC2 activation at least in glioma cells. Our evidence include that Tspan8 forms a complex with mTORC2 component rictor in both human glioma tissues and cells. Further, Tspan8 knockdown inhibited mTORC2 mTOR-rictor assembly as well as AKT (Ser-473) and PKCα phosphorylation. Interestingly, Tspan8 silencing did not affect mTORC1 assembly (mTOR-Raptor association) or activation (indicated by phosphorylation of S6K1) in glioma cells. AKT Thr-308 phosphorylation was also intact in Tspan8-depleted glioma cells. Thus, we propose that Tspan8 is in complex with rictor-mTOR, which might be important for mTORC2 integrity and activation in glioma cells. Although much still need to be explored, these results indicated a possible unique function of Tspan8 in mTORC2 activation.

## 4. Experimental Section

### 4.1. Cell Culture and Reagents

Human glioma cell lines (CGH-5, TJ-899, SGH-44, TJ-905 and U251MG) were purchased from the Chinese Academy of Sciences Cell Bank (Shanghai, China). Glioma cells were maintained in a 37 °C, 5% CO_2_ incubator in DMEM/RPMI supplemented with 8%–10% fetal bovine serum (FBS) and were routinely passaged at 2- to 4-day intervals. Antibodies to rictor (sc-81538), integrin α3 (sc-374242), Tspan8 (sc-169702), AKT1 (sc-5298), mTOR (sc-8319), p-protein kinase C α (PKCα) (Ser-657, sc-12356), PKCα (sc-208) and Glyceraldehyde-3-phosphate dehydrogenase (GAPDH, sc-32233) were purchased from Santa Cruz Biotech (Santa Cruz, CA, USA). P-AKT (Ser-473) antibody (9271), p-AKT (Thr-308) antibody (9275), and p-p70 S6 kinase 1 (p-S6K1, Thr-398) (9209) antibody were obtained from Cellular Signaling Tech (Beverly, MA, USA).

### 4.2. Western Blot Analysis

Cells and glioma tissues were lysed in lysis buffer (30 mM Tris-HCl pH 8.0, 150 mM NaCl, 1% NP-40, 1 mM phenylmethylsulfonylfluoride and protease inhibitor cocktail) on ice for 30 min centrifuged at 18,000× *g* for 15 min at 4 °C, and the supernatants were collected as the cell lysates. Equal amounts of protein (20–30 μg) from each sample were separated by SDS-PAGE and transferred to PVDF membranes. Following incubation of indicated primary antibodies and secondary antibodies, specific bands were visualized using enhanced chemiluminescence (ECL) reagents (Amersham Pharmacia Biotech, Piscataway, NJ, USA) based on the molecular weight (*M*w). Band intensity was always quantified through total gray using the ImageJ software (NIH, Bethesda, MD, USA).

### 4.3. Co-Immunoprecipitation (IP)

Aliquots of 1000–1200 µg of proteins of glioma tissues or glioma cells in 1 mL IP lysis buffer (containing 3% CHAPS) (see [20]) were pre-cleared by incubation with 30 µL of protein A/G Sepharose (beads) (Sigma, Shanghai, China) for 1 h at 4 °C. The pre-cleared samples were incubated with indicated antibody overnight at 4 °C. Thereafter, thirty µL of protein A/G beads were added, and the samples were incubated for 2 h at 4 °C. The beads were washed five times with PBS (4 °C) and once with the lysis buffer, boiled, separated by 10% SDS-PAGE, and transferred onto a PVDF membrane followed by Western blotting testing indicated proteins.

### 4.4. Transwell Assay

U251MG cells (1 × 10^6^) were seeded into the upper compartment (Costar). Each polycarbonate filter had been coated with 10 µL of 0.5% Matrigel before the addition of cells. After 18 h of incubation at 37 °C in 5% CO_2_, the cells on the underside of the chamber were fixed, stained, and photographed. The five visual fields were photographed in every membrane, with manual counting of stained cells. All samples were run in triplicate.

### 4.5. MTT Cell Viability Assay

Cell viability was determined by 3-(4,5-dimethylthiazol-2-yl)-2-5-diphenyltetrazolium bromide (MTT) cell viability assay [35,36]. Ten μL MTT reagent (5 mg/mL) (Sigma Chemical Co., St. Louis, MO, USA) was added per well for 4 h at 37 °C. Next, 150 μL detergent solution was added to lyse the cells and solubilize colored crystals. Plates were then incubated in the dark for 1 h. Optical density (OD) was obtained using micro-plate reader (Molecular Devices, Sunnyvale, CA, USA) at 490 nm wavelength. All samples were run in triplicate.

### 4.6. siRNA

U251-MG cells were seeded into six-well plates at 2 × 10^5^ cells per well, grown for 24 h in complete medium and then transfected with ON-TARGET plus Smart pool siRNA specific to Tspan8 [-1(J-010219-05-0002)/-2(J-010219-06-0002)], rictor [-1(J-016984-06-0002)/-2(J-016984-07-0002)], integrin α3 (l-004571-00-0005), or scrambled siRNA (scRNAi, d-001810-01-05)-negative control, all designed and purchased from Dharmacon (Chicago, IL, USA), at a final concentration of 100 nM using Hyperfect transfection reagent (Qiagen, Courtaboeuf Cedex, France) according to the manufacturer’s directions. To exam RNAi efficiency, cells were harvested 2 days after siRNA transfection and analyzed by Western blots as described above.

### 4.7. Patient’s Glioma Tissues Isolation and Preparation

Surgery-isolated human glioma tissues of high-grade (3–4) and their surrounding normal tissues were homogenized and lysed, proteins were isolated, and expression and association of list proteins in lysed tissues were examined by western blots and Co-IP, respectively. Normal brain tissues were obtained from a stroke patient who underwent brain surgery at author’s institution. The study was approved by the institutional review board of all authors’ affiliations, and written informed consent was obtained from each participating patient. All clinical investigations were conducted according to the Code of Ethics of the World Medical Association (Declaration of Helsinki). The privacy rights of human subjects are always observed.

### 4.8. Statistical Analysis

All statistics were calculated using SPSS 11.0 statistical software (SPSS, Chicago, IL, USA). Descriptive statistics including mean and SD along with one-way ANOVAs were used to determine significant differences. *p* < 0.05 was considered significant.

## 5. Conclusions

In summary, the results of this study suggest over-expressed Tspan8 in malignant glioma forms a complex with rictor and integrin α3 to regulate mTORC2 activation and glioma cell migration. Targeting Tspan8-rictor-integrin α3 complex may provide a potential therapeutic intervention for malignant glioma.
